# COX inhibitors and breast cancer

**DOI:** 10.1038/sj.bjc.6602942

**Published:** 2006-01-17

**Authors:** D Mazhar, R Ang, J Waxman

**Affiliations:** 1 Department of Cancer Medicine, Division of Medicine, Faculty of Medicine, Imperial College London, Room 1014 Garry Weston Centre, Hammersmith Campus, Du Cane Road, London W12 0NN, UK

**Keywords:** cyclo-oxygenase (COX), prostaglandin, breast cancer, NSAIDS, selective COX-2 inhibitor

## Abstract

There is considerable evidence to suggest that prostaglandins play an important role in the development and growth of cancer. The enzyme cyclo-oxygenase (COX) catalyses the conversion of arachidonic acid to prostaglandins. In recent years, there has been interest in a possible role for COX inhibitors in the prevention and treatment of malignancy. Cyclo-oxygenase-2 (COX-2) is overexpressed in several epithelial tumours, including breast cancer. Preclinical evidence favours an antitumour role for COX inhibitors in breast cancer. However, the epidemiological evidence for an association is conflicting. Trials are being conducted to study the use of COX inhibitors alone and in combination with other agents in the chemoprevention of breast cancer, and in the neo-adjuvant, adjuvant, and metastatic treatment settings. In evaluating the potential use of these agents particularly in cancer chemoprophylaxis, the safety profile is as important as their efficacy. Concern over the cardiovascular safety of both selective and nonselective COX-inhibitors has recently been highlighted.

Breast cancer is the most common malignancy in women in industrialised nations and the second leading cause of female cancer-related mortality. Approximately 40 000 women develop breast cancer in the UK each year. The incidence of breast cancer has increased by two-thirds over the last 15 years. Mortality rates though have fallen by one-third, and this is likely to be due to earlier detection of breast cancer because of screening, and the increased use of adjuvant therapies. In recent times, the prospect of further improvements in mortality rates has grown with hope provided by new chemotherapy agents and monoclonal antibody therapy directed at cell surface molecules. But can we do even better for women with breast cancer using cheap and simple treatments that are in current use?

Nonsteroidal anti-inflammatory drugs (NSAIDs) are a group of widely available, inexpensive medicines. The analgesic, anti-inflammatory, antipyretic and antithrombotic effects of salicylate in willow bark and other plant extracts were recognised in ancient Egypt and Greece. These properties have been extensively exploited in numerous fields of clinical medicine since the 19th century, and in cancer patients primarily for analgesia.

Nonsteroidal anti-inflammatory drugs inhibit the enzyme cyclo-oxygenase (COX), which catalyses the conversion of arachidonic acid to prostaglandins (PGs). Prostaglandins are important mediators of signal transduction pathways and are involved in cellular adhesion, growth and differentiation. In recent years, interest has been aroused in a possible role for aspirin and other NSAIDs in the prevention of malignancy. The most persuasive evidence to date relates to colorectal cancer. Meta-analyses of observational studies suggest that NSAIDs reduce the risk of colorectal cancer by around a half (Garcia Rodriguez and Huerta-Alvarez, 2000). For this reason, the US Food and Drug Administration has approved the use of the selective COX-2 inhibitor, celecoxib, in the prevention of colorectal polyps in patients with familial adenomatous polyposis.

It has been suggested that there is a possible role for COX inhibitors in the chemoprevention, and possibly even treatment of breast cancer. In this article we review the current experimental, epidemiological and clinical evidence available on the possible link between COX and breast cancer, coming to a consensus as to whether COX-inhibition is a worthwhile potential strategy in the prevention and treatment of breast cancer.

## COX AND BREAST CANCER: MOLECULAR RELATIONSHIP

### The biochemistry of COX

NSAIDs inhibit the cyclo-oxygenase enzymes 1 and 2, the rate-limiting enzymes in the conversion of arachidonic acid to prostaglandins. The two COX isoforms have distinct tissue distributions and physiological functions. Cyclo-oxygenase-1 is constitutively expressed in many tissues and cell types, whereas the inducible isoenzyme COX-2 is pro-inflammatory in nature and expressed only in response to certain stimuli such as mitogens, cytokines, growth factors, or hormones. Specific COX-2 inhibitors have been developed, and these largely avoid the gastrointestinal side effects associated with NSAID use, which are thought to be due mainly to COX-1 inhibition. Prostaglandins are important mediators of signal transduction pathways and are therefore involved in cellular adhesion, growth and differentiation.

### COX, prostaglandins and breast cancer

There is a clear relationship between tissue prostaglandin levels in human breast tumours, the development of metastases and survival (Bennett, 1986). The main product of COX-2, prostaglandin E_2_, is synthesised by several human breast cancer cell lines and is found at high levels in tumour cells (Screy and Patel, 1995). High concentrations of prostaglandin E_2_ have been associated with risk of metastases and a lack of oestrogen and progesterone receptors (Fulton and Heppner, 1985).

COX-2 is overexpressed in breast cancer cell lines such as the highly invasive, metastatic line MDA-MB-231 (Liu and Rose, 1996) as well as in tumours. In one study, COX-2 expression was detected by PCR in 13 human breast tumours with no detectable expression in normal breast tissue (Parrett *et al*, 1997). A correlation was also observed between COX-2 expression and increasing tumour cell density. Contrasting findings come from a series of 44 cases where COX-2 protein was detected in just two patients (Hwang *et al*, 1998).

Conclusions become clearer when larger numbers of patients' tumours are examined. In an immunohistochemical study of 1576 invasive breast carcinomas, there was moderate to strong COX-2 expression in 37% of the samples (Ristimaki *et al*, 2002). This observation, which has been replicated in other studies involving large patient numbers, should be regarded as definitive, and the evidence from past studies should be disregarded because of the small sample size.

COX staining is not specific to malignant cells but also detectable in premalignant breast tissue. A higher frequency of COX-2 was expressed in ductal carcinoma *in situ* than invasive breast cancer, suggesting that COX-2 may have a role in preinvasive disease. However, all is not as straightforward as it might first appear. Boland *et al* (2004) found that there was no significant difference in COX-2 expression, comparing normal breast tissue from reduction mammoplasty and normal breast tissue surrounding ductal carcinoma *in situ*, and also no difference in COX-2 expression between ductal carcinoma *in situ* and invasive breast cancer.

### COX-2 and breast cancer progression

COX-2 expression is correlated with prognostic markers that reflect a poor chance for survival, which includes tumour size, axillary node metastases, tumour grade, ductal histology, receptor negative disease and HER-2 amplification (Ristimaki *et al*, 2002; Boland *et al*, 2004). Moreover, elevated COX-2 expression has recently been shown to correlate with distant metastases in breast cancer (Ranger *et al*, 2004).

**Figure 1:**
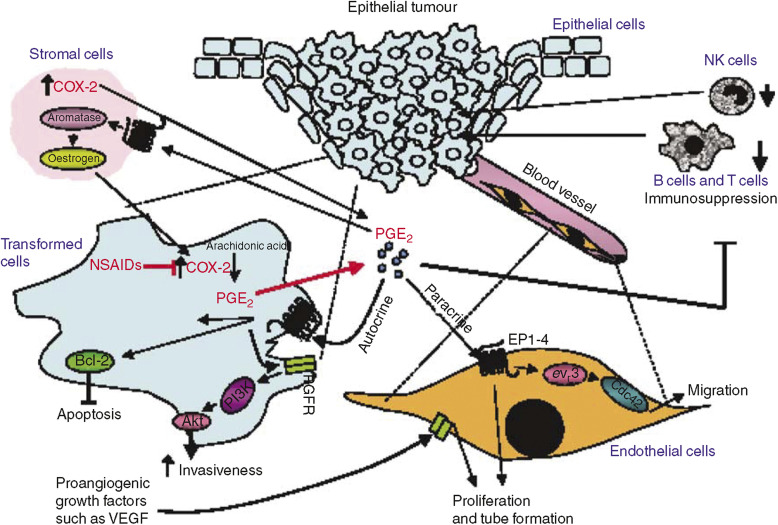
Mechanisms by which COX-2 and PGE2 could modulate mammary tumour development. In epithelial tumours of the mammary gland, COX-2-derived PGE2 may stimulate proliferation and angiogenesis, enhance invasiveness, protect cells from apoptosis, and modulate immunosuppression. Solid malignancies are composed of multiple types of cells, which produce signals that work in both a paracrine and autocrine manner as depicted. COX-2, cyclooxygenase-2; NK, natural killer; EGFR, epidermal growth factor receptor; NSAIDs, nonsteroidal anti-inflammatory drugs; VEGF, vascular endothelial growth factor; PGE2, prostaglandin E2. Reprinted from Wang D, Raymond ND. Cyclooxygenase-2: a potential target in breast cancer. Semin Oncol 2004; 31 (Suppl 3): 64–73

COX-2 is related to cancer outlook through direct and indirect mechanisms. Prostaglandins may directly stimulate mitogenesis through a direct effect on fibroblasts, osteoblasts, and mammary cells. Cyclo-oxygenase-2 indirectly affects mutagenesis, angiogenesis, and increased cell migration and apoptosis (Figure 1). Celecoxib has been shown to inhibit proliferation of human breast cancer cell lines (Arun *et al*, 2001).

The combination of COX-2 inhibitor with standard cancer chemotherapeutic and/or radiation may provide additional therapeutic paradigms in the treatment of various human cancers.

## COX-2 AS A POTENTIAL TARGET FOR PREVENTION AND TREATMENT OF BREAST CANCER

Translational experiments in animal models link COX with breast cancer. Transgenic mice with the COX-2 gene inserted under the control of the mouse mammary tumour virus promoter developed mammary tumours after several cycles of pregnancy and lactation while virgin animals remain tumour free (Liu *et al*, 2001). This provides evidence that overexpression of COX-2 itself is sufficient to induce tumorigenesis, but of potentially greater clinical significance is the evidence that if the transgenics were given a COX-2 inhibitor, mammary tumorigenesis was repressed (Narko *et al*, 2005).

In another study using nonselective COX inhibitors, a 35-day course of ibuprofen administered to rats with carcinogen-induced mammary tumours, led to a significant reduction in tumour volume. The tumours showed reduced expression of both COX isoforms (Robertson *et al*, 1998).

Specific COX-2 inhibitors can prevent mammary tumours from developing in experimental animals. Nimesulide reduced the size and numbers of carcinogen-induced tumours (Nakatsugi *et al*, 2000) and celecoxib inhibited the development of carcinogen-induced mammary tumours (Abou-Issa *et al*, 2001). Celecoxib has also been showed to significantly delay the onset of HER2/neu-induced tumours (Howe *et al*, 2002). HER2/neu-induced mammary tumours and angiogenesis have been shown to be reduced in COX-2 knockout mice (Howe *et al*, 2005).

It has been demonstrated that PGE2 stimulates aromatase transcription leading to increased concentrations of oestrogens (Harris *et al*, 1999). Overexpression of COX-2 in breast cancer may lead to increased PGE2 synthesis and this in turn to progression of oestrogen-dependent disease. Therefore, inhibition of PGE2 by COX-2 inhibitors may inhibit aromatase activity and when combined with aromatase inhibitors reduce tumours by inhibiting a common target. Indeed, there is preclinical data from a rodent model to suggest that celecoxib when combined with exemestane significantly inhibits the growth of mammary tumours (Pesenti *et al*, 2001).

The antitumorigenic effects of NSAIDs and selective COX-2 inhibitors may involve other mechanisms than COX-2 inhibition: for example, high concentrations of NSAIDs or selective inhibitors of COX-2 suppress the growth of cells in culture that do not express COX-2 (Hwang *et al*, 2002). Moreover, a recent clinical trial found that low-dose aspirin, which has virtually no COX-2 inhibitory effects, had a chemoprotective effect in individuals at increased risk of developing colorectal cancer (Huls *et al*, 2003).

## EPIDEMIOLOGY OF COX INHIBITOR USE AND BREAST CANCER INCIDENCE

So, the balance of evidence from cell lines and animal models support the notion that COX-2 may be involved in breast carcinogenesis, and that COX-2 inhibition could play a preventative or even a therapeutic role. But what is fact in cell line and animal models of cancer may be fiction in tumours. What is the epidemiological evidence for a possible link between COX-2 inhibition and breast cancer?

A recent meta-analysis (Gonzalez-Perez *et al*, 2003) of nine case–control studies and seven cohort studies showed a slight but significant reduction of breast cancer incidence among users of aspirin and other non-aspirin NSAIDs. There was significant heterogeneity of results, which is largely explained by the differences in study designs, exposure assessment, and to a lesser degree, the inclusion of lag time analysis.

A meta-analysis (Khuder and Mutgi, 2001) of eight case–control and six cohort studies examined the effect of dose and frequency of NSAID use on risk reduction. Only two studies provided evidence of significant trend of risk reduction with increasing exposure to NSAIDs (Coogan *et al*, 1999; Sharpe *et al*, 2000), but there were insufficient data to estimate the overall combined dose–response effect for either duration or frequency of use in the meta-analysis.

A recent large prospective cohort analysis attempts to answer the question of whether increasing frequency and duration of use of NSAIDs is associated with a reduced risk of breast cancer (Harris *et al*, 2003). The US Women's Health Initiative Observational Study of 80 741 postmenopausal women between the ages of 50 and 79 years, who were followed-up for an average of 43 months after baseline interview included an assessment of breast cancer risk factors and NSAID use. There were 1392 confirmed cases of breast cancer. Regular NSAID usage of two or more tablets a week for 5–9 years produced a 21% reduction in breast cancer incidence; NSAID usage for over 10 years produced a 28% reduction and there was statistically significant inverse linear trend of breast cancer incidence with the duration of NSAID use. The estimated risk reduction was greater with ibuprofen than with aspirin. Subgroup analysis of breast cancer risk factors did not result in effect modification. Regular use of low-dose aspirin and acetaminophen was unrelated to the risk of breast cancer.

This result is at odds with the findings of another large study involving 734 899 women in a nested case–control design. This study, using the General Practice Research Database, found a protective role for aspirin and paracetamol if taken for one year or longer, with daily doses of aspirin and paracetamol showing greatest risk reduction. There was no risk reduction for use of non-aspirin NSAIDs (Garcia Rodriguez and Gonzalez-Perez, 2004).

The dose–response relationship shown by the US Women's Health Initiative Observational Study was also illustrated in a population-based case–control study involving 1442 cases and 1420 controls, comparing aspirin, ibuprofen and acetaminophen. Here there was a statistically significant inverse association for any NSAID use, and frequent use. For aspirin use, the effects for frequency of use were stronger than duration of use. This differs with ibuprofen use where frequency and duration of use was not associated with decreasing risk. Acetaminophen was unrelated to the incidence of breast cancer (Terry *et al*, 2004). The authors examined the relationship between the observed risk reduction of breast cancer in patients taking NSAIDs and hormone receptor status. They concluded that there was a reduction in risk and this was mainly of hormone receptor-positive tumours. This result leads credibility to the theory that the COX-2 inhibitors' action is through aromatase inhibition. However, this is the only epidemiological study so far to show that the mechanism of NSAID activity is through hormone receptor, and needs to be replicated before the findings can be accepted as conclusive.

## CLINICAL TRIALS INVOLVING COX-2 INHIBITORS AND BREAST CANCER

Trials are being conducted to study the use of celecoxib alone and in combination with other agents in chemoprevention, and in the neo-adjuvant, adjuvant, and metastatic treatment settings. These are mostly small efficacy safety studies and have so far only reported on safety profile (Banu and Goss, 2004). For example, a prospective pilot study recruited 32 patients to study the use of FEC (5FU 500 mg m^−2^, Epirubicin 75 mg m^−2^, cyclophosphamide 500 mg m^−2^) and celecoxib (400 mg bd) as a neo-adjuvant treatment for locally advanced cancer. A total of 16 patients each were recruited to two arms (FEC alone and FEC together with celecoxib). The stated end points were clinical, pathologic responses and tolerability. The clinical and pathological responses for the combined treatment arms were 81.3 and 87.5, respectively, *vs* 62.5 and 62.5% in the single treatment arm. The regimens were well tolerated with no significant clinical cardiac toxicity.

A phase II randomised trial of trastuzumab, with or without celecoxib, in a series of 12 patients with metastatic breast cancer who had previously progressed after trastuzumab-based treatments, found that there was no treatment effect, although the drug combination was well tolerated (Dang *et al*, 2004). This study consisted only of patients who had been pretreated with trastuzumab. The effect on treatment-naive patients is still unknown and being investigated in an ongoing trial. Results of a randomised phase II study in 111 postmenopausal women with advanced breast cancer treated with exemestane and celecoxib indicated a longer time to progression with no additional side effects from the use of the combination (Dirix *et al*, 2003).

The high expression of COX-2 in ductal carcinoma *in situ* (DCIS) has led to interest in the use of COX-2 inhibitors in this clinical setting. Studies are now looking at the effect of celecoxib on both oestrogen (ER) negative and positive DCIS. In addition, trials have been proposed using adjuvant celecoxib in ER-negative DCIS to determine whether it prevents recurrence after wide local excision.

## LONG-TERM SAFETY OF COX INHIBITION

Even antiplatelet doses of aspirin cause increase in gastrointestinal and intracerebral bleeding, especially with prolonged treatment of large numbers of healthy people. Evidence is now emerging about the safety of specific COX-2 inhibitors. Earlier studies seem to confirm improved gastrointestinal tolerance compared with conventional NSAIDs (Bombardier *et al*, 2000; Silverstein *et al*, 2000). However, there were worries about a possible prothrombotic effect of COX-2 inhibitors (Bombardier *et al*, 2000) and the recent withdrawal of rofecoxib due to increased cardiovascular thrombotic risk in the Adenomatous Polyp Prevention on Vioxx (APPROVe) trial has highlighted new safety concerns about this class of drugs. The safety of celecoxib is currently being examined following results from the Adenoma Prevention with Celecoxib (APC) trial, which found patients taking 400 to 800 mg day^−1^ of celecoxib had a 2.5- to 3.4-fold increased risk of major fatal or non-fatal cardiovascular events *vs* placebo. The use of celecoxib in this trial has now been suspended. The cardiovascular safety of conventional NSAIDs has also been recently questioned (Hippisley-Cox and Coupland, 2005).

A joint meeting of the American Arthritis Advisory Committee and the Drug Safety and Risk Management Advisory Committee was convened in early 2005 to review the safety of COX-2 inhibitors. The committee voted unanimously that all of the COX-2 inhibitors currently (or previously) available in the United States (celecoxib, valdecoxib, rofecoxib) significantly increase the risk of cardiovascular events in users of these drugs. Considering potential benefits as well as risks and their magnitude, the committee voted unanimously in favour of keeping celecoxib on the market for its current indicated uses. Most panelists favoured restrictions on direct-to-consumer advertising of COX-2 inhibitors. All voted in favour of requiring future agents in this class (both COX-2 and nonselective NSAIDs) to perform cardiovascular safety studies prior to market introduction.

## CONCLUSION

So where does this leave us in assessing the role of COX-2 inhibition for the prevention and treatment of breast cancer in the clinical setting? Certainly, there is little evidence at present that these drugs are effective treatments for established breast cancer. Work is currently in progress to investigate the possible role of COX inhibitors in limiting the development of invasive breast cancer from ductal carcinoma-*in situ*. Also, clinical trials are being conducted to study the use of specific COX-2 inhibitors both alone and in combination with other agents in early and advanced breast cancer.

There seems to be a small but potentially significant protective role of NSAIDs on breast cancer risk. It is not clear if aspirin use is associated with a different risk reduction compared with other NSAIDs and, indeed, selective COX-2 inhibitors and questions remain over drug dosage and patient selection. Table 1 summarises the available data for an association between COX and breast cancer.

While several mechanisms have been suggested for the anticancer action of COX inhibitors, it remains unclear which is the most important and, indeed, whether inhibition of COX-2 is the sole reason for the effects observed in this context. Also, if there is a protective role to be played by COX inhibitors in breast cancer, there are questions of how much drug should be taken and for how long. Concern over the long-term safety of conventional NSAIDs and selective COX-2 inhibitors has recently been highlighted. In evaluating the potential use of NSAIDS in cancer chemoprophylaxis, the safety profile of these drugs is as important as their efficacy.

On the face of it, at the end of this review, the reader has probably been left with more questions than there are answers. However, if the use of aspirin and other NSAIDs is associated with reduced incidence of breast cancer, this could have a major public health impact. Work is in progress to detail the possible link of COX-2 inhibition and breast cancer, and also to assess the long-term safety and hence viability of this potentially valuable and viable chemopreventative approach.

## Figures and Tables

**Table 1:** Overview of studies exploring the association of COX and breast cancer

**Author(s)**	**Type of study**	**Finding**
Screy and Patel (1995)	*In vitro*	PGE2 is synthesised by breast cancer cell lines
Liu and Rose (1996)	*In vitro*	COX-2 is overexpressed in MDA-MB-231 breast cancer cells
Ristimaki *et al* (2002)	*In vitro*	COX-2 expression is found in 37% of invasive breast cancers
Arun *et al* (2001)	*In vitro*	Celecoxib inhibits growth of human breast cancer cell lines
Liu *et al* (2001)	Preclinical/*in vivo*	COX-2 overexpression induces mammary tumorigenesis in transgenic mice
Narko *et al* (2005)	Preclinical/*in vivo*	COX-2 inhibitors reduce mammary tumorigenesis in COX-2 transgenic mice
Abou-Issa *et al* (2001)	Preclinical/*in vivo*	Celecoxib inhibits development of carcinogen-induced mammary tumours
Howe *et al* (2005)	Preclinical/*in vivo*	HER-2/neu-induced mammary tumours and angiogenesis are reduced in COX-2 knockout mice
Gonzalez-Perez *et al* (2003)	Epidemiological/meta-analysis of nine case–control and seven cohort studies	Aspirin and NSAIDs significantly reduce breast cancer risk
Khuder and Mutgi (2001)	Epidemiological/meta-analysis of eight case–control and six cohort studies	Increasing exposure to NSAIDs does not significantly reduce breast cancer risk
Harris *et al* (2003)	Epidemiological (cohort)	Regular NSAID use reduces breast cancer incidence; there is an inverse linear trend of breast cancer incidence with NSAID use duration
Garcia Rodriguez and Gonzalez-Perez (2004)	Epidemiological (nested case–control)	Aspirin use for a year or longer protects against breast cancer development; no risk reduction seen with other NSAIDs
Terry *et al* (2004)	Epidemiological (case–control)	NSAID use (as well as frequency of use) is inversely related to risk of breast cancer
